# Utility of Biogenic Iron and Its Bimetallic Nanocomposites for Biomedical Applications: A Review

**DOI:** 10.3389/fchem.2022.893793

**Published:** 2022-07-01

**Authors:** Ali Abedini, Mojtaba Rostami, Hamid Reza Banafshe, Mehdi Rahimi-Nasrabadi, Ali SobhaniNasab, Mohammad Reza Ganjali

**Affiliations:** ^1^ Young Researchers and Elite club, Central Tehran Branch, Islamic Azad University, Tehran, Iran; ^2^ School of Chemistry, College of Science, University of Tehran, Tehran, Iran; ^3^ International Iberian Nanotechnology Laboratory (INL), Braga, Portugal; ^4^ Department of Pharmacology, School of Medicine, Kashan University of Medical Sciences, Kashan, Iran; ^5^ Chemical Injuries Research Center, Systems Biology and Poisonings Institute, Baqiyatallah University of Medical Sciences, Tehran, Iran; ^6^ Faculty of Pharmacy, Baqiyatallah University of Medical Sciences, Tehran, Iran; ^7^ Institute of Electronic and Sensor Materials, TU Bergakademie Freiberg, Freiberg, Germany; ^8^ Physiology Research Center, Institute for Basic Sciences, Kashan University of Medical Sciences, Kashan, Iran; ^9^ Core Research Lab, Kashan University of Medical Sciences, Kashan, Iran; ^10^ Center of Excellence in Electrochemistry, School of Chemistry, College of Science, University of Tehran, Tehran, Iran; ^11^ National Institute of Genetic Engineering and Biotechnology (NIGEB), Tehran, Iran

**Keywords:** green method, Fe nanoparticles, drug delivery, antibacterial, anticancer

## Abstract

Nanotechnology mainly deals with the production and application of compounds with dimensions in nanoscale. Given their dimensions, these materials have considerable surface/volume ratios, and hence, specific characteristics. Nowadays, environmentally friendly procedures are being proposed for fabrication of Fe nanoparticles because a large amount of poisonous chemicals and unfavorable conditions are needed to prepare them. This work includes an inclusive overview on the economical and green procedures for the preparation of such nanoparticles (flower, fruits, tea, carbohydrates, and leaves). Pure and bimetallic iron nanoparticles, for instance, offer a high bandwidth and excitation binding energy and are applicable in different areas ranging from antibacterial, anticancer, and bioimaging agents to drug delivery systems. Preparation of nano-sized particles, such as those of Fe, requires the application of high quantities of toxic materials and harsh conditions, and naturally, there is a tendency to develop more facile and even green pathways (Sultana, Journal of Materials Science & Technology, 2013, 29, 795–800; Bushra et al., Journal of hazardous materials, 2014, 264, 481–489; Khan et al., Ind. Eng. Chem. Res., 2015, 54, 76–82). This article tends to provide an overview on the reports describing green and biological methods for the synthesis of Fe nanoparticles. The present review mainly highlights selenium nanoparticles in the biomedical domain. Specifically, this review will present detailed information on drug delivery, bioimaging, antibacterial, and anticancer activity. It will also focus on procedures for their green synthesis methods and properties that make them potential candidates for various biomedical applications. Finally, we provide a detailed future outlook.

## 1 Introduction

Preparation of magnetic or non-magnetic nanomaterials through reduction of metal salts, thermal decomposition, and metal evaporation has been a hot topic during the recent years, due to their unique physical/chemical properties as opposed to the corresponding bulk forms. Consequently, a wide range of applications of such materials in bioseparation and biomedical procedures, biosensors, cytotoxicity, antibacterial, catalysts, magnetic resonance imaging (MRI), and environmental remediation are increasing alarmingly (Rahimi-Nasrabadi et al., 2015; Rahimi-Nasrabadi et al., 2016; Abamor et al., 2017; Naderi et al., 2017; Rostami, 2017; Sobhani-Nasab et al., 2017; Eghbali-Arani et al., 2018a; Amani et al., 2018; Eghbali-Arani et al., 2018b; Padash et al., 2018; Pourmasoud et al., 2018; Sedighi et al., 2018; Gandomi et al., 2019; Sobhani-Nasab et al., 2019; Nayak et al., 2022; Rostami et al., 2022; Wang et al., 2022).

Various research teams have reported the synthesis of mono- and bimetallic nanoparticles of iron through chemical reduction reactions using diverse polymers and organic solvents (Wang and Zhang, 1997; Ponder et al., 2000; Sun et al., 2000; Saleh et al., 2005; Peng et al., 2006). Also, a wide range of plant extracts, carbohydrates, peptides, proteins, organic solvents, oligonucleotides, lipids, dendrimers, phospholipids, surfactants, and polymers have been reportedly used as stabilizers to control the shape of noble metal nanoparticles (Murphy et al., 2005; Khullar et al., 2012; Bashir et al., 2014; Murray et al., 2016). Lin et al. (2001) reported a reverse micelle procedure based on the application of 1-butanol, octane, and cetyltrimethylammonium bromide to prepare iron nanoparticles coated with a gold film. In another work, He and Zhao (2005) used solutions of starch in water for stabilizing the potentials in a procedure for preparation of iron nanoparticles using a mixed solution of Fe^2+^ and Fe^3+^ in water using sodium borohydride (NaBH_4_). There is a general trend toward green environmentally friendly chemical technologies, in the light of the global environmental crises. This concern is reflected by the publication of various green chemistry books in the past decade (Ahluwalia, 2009; Anastas and Li, 2012) covering general and specialized procedures like ultrasound and microwave approaches as well as green synthesis, analysis, tribology, polymerization, engineering, and manufacturing. Furthermore, environmentally friendly techniques for production of food, textiles, hydrogen and syngas, biocomposites, particle technology, biomass, wastewater treatment, and biofuels have been covered in different references (Allen and Shonnard, 2001; Chen et al., 2011; Mathers and Meier, 2011; Mulvaney, 2011; Puigjaner, 2011; Boye and Arcand, 2012; de la Guardia and Garrigues, 2012; Kan, 2012; Lee and Henthorn, 2012; Lee and Shah, 2012; Lofrano, 2012; Nosonovsky and Bhushan, 2012; Schiebl, 2012; Siyamak, 2012; Debalina and Pike, 2017). Chemists all over the world prescribe the 12 principles of green chemistry in their works (Anastas and Warner, 1998; Singh et al., 2021). It is a fact that various methods, such as aerosol technologies, UV irradiation, laser ablation, lithography, photochemical reduction, and ultrasonic procedures, have proven to be applicable for production of nanoparticles. The economic and environmental costs of these techniques are still high, and therefore an ongoing interest exists to develop sustainable eco-friendly procedures for the purpose mentioned previously (Narayanan and Sakthivel, 2010).

On the other hand, evaluation of the negative effects of nanomaterials is still a relatively hot topic with lots of unanswered questions, and it is believed that the preparation of these materials through using eco-friendly and biocompatible reagents can lessen the known and unknown adverse effects of these materials on humans and the environment (Varma, 2012). Non-toxic solvents (ideally water), sealed reactors, eco-friendly procedures not requiring contacting reaction media and air (microwave, ultrasound, biological methods, magnetic, and hydrothermal, among others), and low-temperature processes are among the solutions. This review also focuses on the eco-friendly methods for preparation of nanoparticles and nanomaterials using green reducing and capping agents like herbal extracts (Gan and Li, 2012). Furthermore, natural materials like vitamins, sugars, biodegradable polymers and microorganisms and herbal extracts, among the rest of these reducing and capping agents, have proven to be suitable for large-scale applications (Iravani, 2011).

The main chemical agent in some reactions has been found to be polyphenols. These methods are simple, cost-effective, and rather reproducible and produce stable products (Kalaiarasi et al., 2010). It is also possible to use microorganisms for production of nanoparticles. Yet, the methods are rather slow and produce limited particle sizes and shapes as compared with the method based on herbal extracts. Fungi extracts are currently finding worldwide popularity as tools for use in the synthesis of nanoparticles (Dhillon et al., 2012). Biomaterials generally act as eco-friendly through eliminating the need for using toxic chemical agents or harsh conditions (Parsons et al., 2007). The mechanisms of the corresponding bioreduction reactions (Xie et al., 2007a; Zhou et al., 2010; Huang et al., 2011) and those of the catalytic reactions of the products of these reactions have been evaluated (Vilchis-Nestor et al., 2009; Du et al., 2011; Zhan et al., 2012).

**FIGURE 1:**
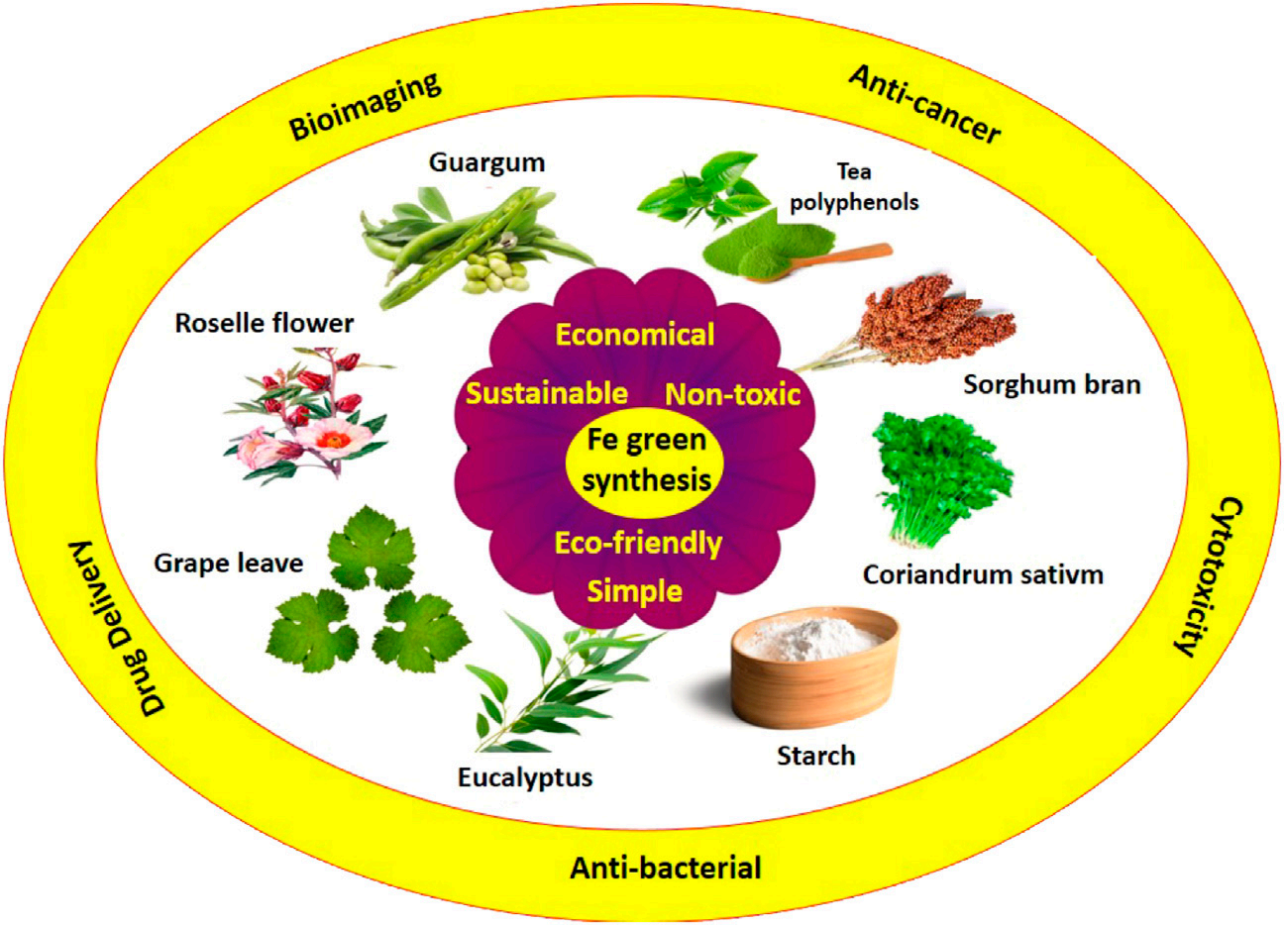
Green capping agent used for the preparation of Fe nanoparticles.

It is noteworthy that although the application of these green routes is gaining momentum, it is still not as widespread as classical wet-chemistry procedures (Kharissova and Kharisov, 2008; Kharisov et al., 2012a), while they have important advantages like very near to zero contaminations. Even when wastes are left or produced (except for the case of microbial reactions), they are compatible with the environment and living creatures due to their origin. Another advantage of these methods is the rather low-cost precursors (Abdullah et al., 2020). These can change the methods to interest substitutes in many conventional techniques. In this review, the major focus is laid on the biomedical domain of iron nanoparticles by covering their utilities in cancer therapy, drug delivery, bioimaging, and antibacterial applications. Figure 1 shows the schematic representation of the methods for the green synthesis of Fe nanoparticles and their biomedical applications.

## 2 Preparation of Fe Nanoparticles and Its Biometallic Through Plants

Reduction of metal ions and the stability of their complexes during the nucleation and growth phases, leads to the formation of metal oxide nanoparticles. Extracts of seeds, flowers, leaves, and green plants, are known to contain high quantities of proteins, amino acids, polyphenols, and reducing sugars (Nejati et al., 2022). These materials can act as reduction and capping agents (Besbes et al., 2004; Xie et al., 2007b; Khan et al., 2012; Hussain et al., 2014). Njagi et al. prepared Fe and Ag nanoparticles using vitamin aqueous sorghum bran extracts (Njagi et al., 2010; Abdullah et al., 2020). Verma et al., used tea polyphenols (*Camellia sinensis*) to prepare metallic Fe nanoparticles at an ambient temperature without using surfactants or polymers as stabilizers (Hoag et al., 2009; Nadagouda et al., 2010). Khan and Al-Thabaiti (2018) reported a biomimetic method for formation of Fe nanoparticles using FeCl_3_ and *Hibiscus sabdariffa*, Roselle flower aqueous extract (HBS), as capping and reducing agents. The morphology of green synthesized Fe nanoparticles was found to depend on the extract of Fe^3+^ concentrations and also the reaction time. The average particle diameter was found to be 18 nm. Fang Luo and et al. reported the synthesis of bimetallic Fe/Pd nanoparticles *via* the green method. This single-step approach was based on the application of an aqueous extract of grape leaves for cost-effective and large-scale preparation of Fe/Pd nanoparticles for field remediation. Some herbal extracts have also been used for the synthesis of bimetallic nanoparticles containing Fe (He and Zhao, 2005; Smuleac et al., 2011). The synthesis of iron nanoparticles and their biometallic composites with plants is environmentally friendly, but controlling the morphology and size of nanoparticles can be difficult. For instance, Fe/Pd nanoparticles were prepared using green tea extracts rather than the application of NaBH_4_ which is a common reducing agent. A summary of the several bio-based template media employed in the preparation of Fe nanoparticles is shown in Table 1.

**TABLE 1:** Biological and biogenic syntheses for preparing Fe NPs.

Type of nanoparticles	Green template	Precursor	Particle size	Reference
Fe-based material	Tea polyphenols	FeCl_3_·6H_2_O	300 and 600 nm	Ouyang et al. (2019)
Fe/Zn bimetallic	*Coriandrum sativum* leaf extract	Ferric chloride and zinc nitrate	20 and 60 nm	Sathya et al. (2018)
Pd/Fe	Polyethyleneglycol	FeSO_4_·7H_2_O	60–100 nm	Wang et al. (2015)
Pd/Fe	Starch	FeSO_4_·7H_2_O	60–100 nm	Wang et al. (2015)
Pd/Fe	Guargum	FeSO_4_·7H_2_O	60–100 nm	Wang et al. (2015)
Core (Fe) with shell (Pd, Ag, Pt, and Au)	Aqueous ascorbic acid (vitamin C)	Fe (NO_3_)_3_ H_2_O	5–60 nm	Nadagouda and Varma, (2007)
Fe/Ni	Eucalyptus leaf extract	FeSO_4_ and Ni (NO_3_)_2_	20–50 nm	Weng et al. (2017)
Fe/Pd	Grape leaf aqueous	FeCl_2_ and PdCl_2_	10–100 nm	Luo et al. (2016)

## 3 Pure and Bimetallic Iron Particles

During the past 3 decades, nanoparticles of metallic iron have been widely used in remediation and treatment of polluted water containing organic or inorganic species, due to their favorable surface properties (Zhang et al., 2011). The various morphologies of iron nanoparticles are significantly attractive for water disinfection and remediation of heavy metals from soils (Kharisov et al., 2012b). The main methods used for the preparation of these particles are based on the application of tea extracts on polyphenol or other herbal extracts (Victor, n.d.). Thus, Fe nanoparticles are prepared using tea extracts (Nadagouda et al., 2010).

Iron nanoparticles prepared using green tea leaf extracts may contain oxohydroxide and iron oxide (Shahwan et al., 2011). These particles have been reportedly used as a Fenton-like catalyst for removal of methyl orange (MO) and methylene blue (MB) dyes from water and is found to have fast removal kinetics, with a second-order behavior for MB and close first-order behavior in the case of MO (Rostami et al., 2018; Rostami, 2019; Akbari et al., 2021). The removal efficiency was found to be complete for both dyes over a wide concentration window of 10–200 mg/l. The same method has been used for preparation of nanoparticles of iron alloys. For instance, polymer membranes [e.g., polyacrylic acid-coated polyvinylidene fluoride membrane] containing immobilized reactive nanoparticles (of Fe/Pd and Fe) have been using tea extracts (Smuleac et al., 2011) and are used for the destruction of trichloroethylene (TCE) as a common contaminant.

## 4 Biomedical Applications

Then, in this review, we have focused mainly on the latest studies of applications, including cancer therapy, drug delivery, and antibacterial, of Fe NPs. Table 2 illustrates several metal-based nanomaterials that are utilized in various domains for enormous applications.

**TABLE 2:** Various types of metal-based NPs that are utilized in various fields (Singh et al., 2021).

Nanoparticle	Example	Application
Metal-based NPs	Manganese (Mn), iron (Fe), silver (Ag), gold (Au), platinum (Pt), selenium (Se), zinc (Zn), and others	Therapeutics, bioimaging, electronics, magnetic resonance imaging (MRI), data storage, antimicrobial agent, and textile
Doped metal NPs	Au–CuO, Pt–ZnO, and others	Antimicrobial, drug delivery, sensors, and others
Sulfide-based metal NPs	FeS, CuS, and others	Bioimaging, cancer therapy, drug delivery, and diagnosis
Metal oxide NPs	CeO_3_, ZnO, CuO, and others	Antimicrobial, biomedical, electronics, optical, and detection
Metal–organic frameworks (MOFs)	Zn-MOF and Mn-MOF	Solar cells, super capacitors, fuel cells, sensors, drug delivery, super capacitors, photoelectrocatalysis, and others

### 4.1 Anticancer Effects

The application of nanotechnology in treating cancer involves engineering, pharmaceutical science, and molecule-based imaging.

Given the unique optical, magnetic, or structural characteristics of semiconducting nanoparticles and nanocrystals, they do not offer target neoplasm antigens (biomarkers), when interacting with neoplasm-targeting ligands (e.g., peptides or antibodies). Nanoparticles with the size of 1–100 nm have very high active surface areas to interact with various detection agents, such as radio-isotopic, optical or magnetic, and medicinal (e.g., anticancer), which creates the chances for genetic and molecular bio-marking opportunities specifically for personalized cancer treatment (Nie et al., 2007). Some research have proven the anticancer effects of magnetic nanoparticles (Peymani-Motlagh et al., 2019a; Peymani-Motlagh et al., 2019b). G.F. Goya and et al. studied and experimented internalizing magnetic nanostructures into dendritic cells to determine the location of the particles and the viability of the cultured cells (Rostami et al., 2021a). Fe nanostructures coated with carbon proved to have toxic effects on dendritic cells and left no effects on their viability (Rostami et al., 2021b).

The observations indicated that dendritic cells can incorporate 10-ca magnetic nanoparticles. Magnetic nanoparticles after 1 day incubation were observed to be 200 nm. Our results suggest that loading dendritic cells with properly functionalized magnetic nanostructures could be a promising strategy for improvement of vectorization in cancer diagnosis and treatment (Goya et al., 2008).

Abdullaeva et al. (2012) prepared onion-like carbon-encapsulated iron nanoparticles. The iron particles were face-centered cubic crystals of 20–30 nm size and with 2–10 nm layered onion-like coatings. The cytotoxicity evaluations involved exposing human lung epithelial A549 cells to these particles for 24 h. Nanoparticles of 10, 20, 40, 80, and 160 lg/ml suspensions were used for this purpose, and the cytotoxic effects were evaluated using MTT and XTT assays, and it was found that higher cell viability was possible under the same conditions, i.e., 95% for the Fe–C nanoparticles. The results showed that the viability of the Fe–C sample was 8% more than that reported in the case of Fe–C prepared through arc-discharge by Kratschmer–Huffmann (Goya et al., 2008). Also, it was reported that Fe nanoparticles held the potentials for use as effective chemotherapeutic and bacteriostatic agents for the fast distinction of various cancers (Naderi et al., 2018). The synthesis of iron nanoparticles and their biomedical composites with eco-friendly plants is a great way to eradicate cancer.

#### 4.1.1 Toxicity Mechanism

**FIGURE 2:**
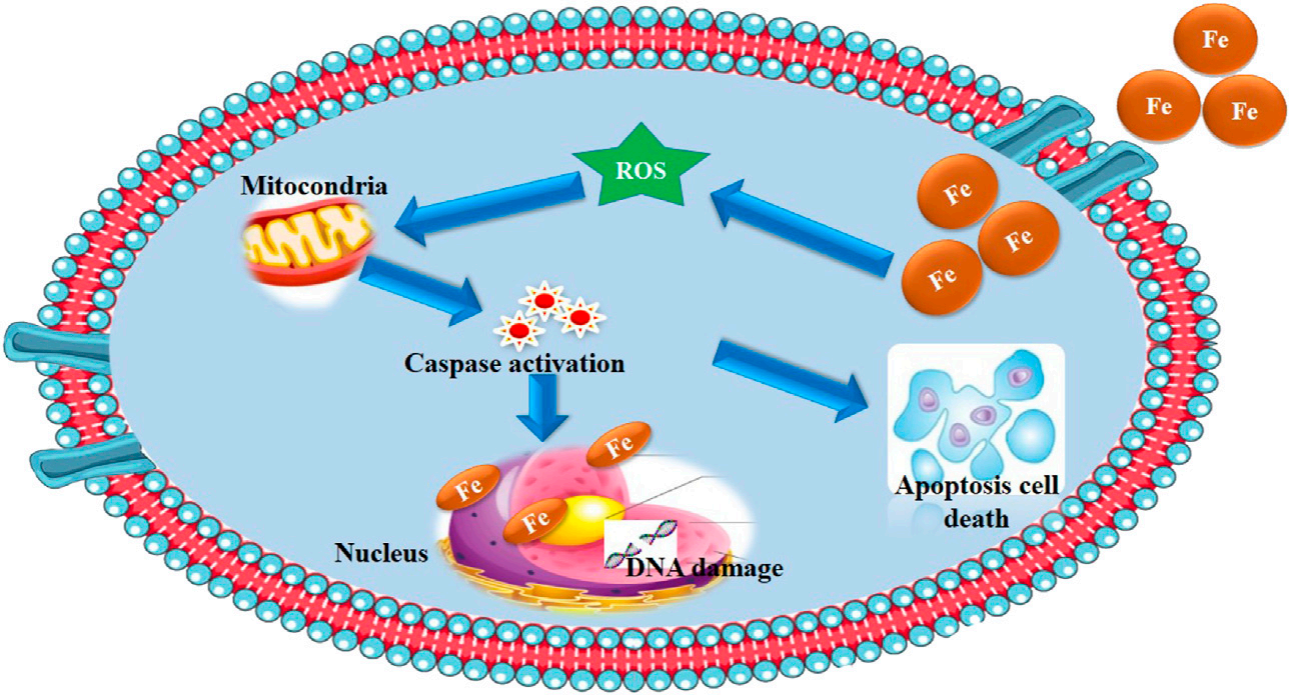
Schematic representation of the mechanism for the cytotoxicity activity of Fe NPs.

Metal nanoparticles have toxic effects mainly due to the oxidative stress they cause in the intercellular space or through their oxidation leading to formation of free ions in the solution. Fe can pass through cell membranes via calcium transport proteins, and nanoparticles find their way to the cytoplasm through endocytosis. The cell toxicity of the nanoparticles is presented in Figure 2.

#### 4.1.2 Oxidative Stress

The main reasons for the toxic effect of nanoparticles are the formation of ROS and the oxidative stress (Yang et al., 2009). It has been reported that the presence of small amounts of metallic species in a cell can give rise to the formation of large amounts of O^2−^, OH^.^, and H_2_O_2_ (ROS) (Dudev and Lim, 2008) through interference with the cellular electron transport chain (Xia et al., 2008).

The main source of intracellular ROS is mitochondria (Xia et al., 2007). It is known that OH radicals (hydroxyl radicals) are the most reactive ROS and oxidize nearly all organelle species (Yamakoshi et al., 2003). The ROS formed in the cell lead to the formation of O^2−^ through surface electron capture by the nanoparticles (De Berardis et al., 2010). Free radicals create a degree of imbalance in the oxidant/anti-oxidant phenomena of the cells through oxidizing lipids, denaturing proteins and nucleic acids, and stimulating an anti-oxidant defense system (Yang et al., 2009).

Nanoparticles can deactivate lysosomes and destroy their membranes if the pH of lysosomes is acidic (Nohl and Gille, 2005), leading to the transfer of nanoparticles’ nuclei and mitochondria (Wang et al., 2012). In the latter case, the deposition of iron nanoparticles onto the membrane enhances the membrane depolarization to disturb transduction of electrons in the inner membrane. This, in turn, leads to the permeability of membranes and formation of reactive oxygen species (Gabbay et al., 2006; Xia et al., 2007).

The presence of reactive oxygen species in lysosomes can ruin DNA double helix or lead to DNA point mutations (Singh et al., 2009), but in mitochondria, the ROS change mitochondrial respiration and apoptosis, damage cellular redox disequilibria, and per-oxidize the cell membrane lipids (Xia et al., 2007). H_2_O_2_ generated in the cytoplasm easily diffuses the mitochondrial membranes and undergoes the Fenton reaction to form OH damaging DNA and leading to cellular death (Xia et al., 2007).

High amounts of intracellular ROS enhance the gene expression of the death receptor nanoparticles (Yang et al., 2009) as well as redox-sensitive signaling paths under average oxidative stress (Nel et al., 2006; He et al., 2007). Inflammatory responses which could be caused by death and cellular fibrosis would initiate as a result of activation of mitogen-activated protein kinase and the signaling cascades of nuclear factor NF-kB (Nel et al., 2006). Nuclear pores which are larger than 50 nm can allow quantum dots (QDs) to enter the nucleus. This can also happen through direct physical injuries of the nucleus membrane. As a result, the quantum dots can directly interact with the DNA of the nucleus (Wang et al., 2012).

#### 4.1.3 Dissolution of Nanoparticles

The presence of free ions‏ through the dissolution of nanoparticles is the second reason for the high toxicity of metallic nanoparticles (Gabbay et al., 2006). Fe NPs’ high concentrations in suspensions or cellular media (Gunawan et al., 2011) catalyze the Fenton reactions leading to the formation of high quantities of OH, which in turn harms lipids, proteins, and nucleic acids (Hartwig, 2013). Nanoparticles which are present in cells can also move in acidic organelle (e.g., lysosomes) or react with acidic materials (e.g., nucleic acids), freeing more Fe^2**+**^‏, and hence, enhancing damages through oxidation (Cuillel et al., 2014).

Evidently, the dissolution of particles depends on their dimensions, surface area, composition, and other physical/chemical properties as well as conditions such as temperature and pH.

The fact that free orbitals of the cation‏ are able to interact with free electron pairs of atoms like N and O present in chelates allows them to interact and deactivate biomolecules and disrupt their normal functions. Fine nanoparticles in the cell can find their way into nuclei through nuclear pores or have access to the nucleus available due to cellular division, inhibiting the transcription and translation phenomena, and damaging the genetic material through interacting proteins of DNA or DNA (Singh et al., 2009). Furthermore, interactions with cellular signal species can further activate signaling cascades (Miller et al., 2010). Dissolved cations can also destabilize or degrade mRNA through interaction with its proteins (Soenen et al., 2010). These free ions‏ play an important role in the homeostasis of cells (Cuillel et al., 2014), and if their concentration exceeds a certain level, this effect can reverse. Dissolution of nanoparticles can increase the local concentration of ion‏s‏ (Xia et al., 2008), increasing the tendency for the influx of ions‏ through‏ calcium ion channels present in the endoplasmic reticulum–plasma membrane (Hoyal et al., 1998). As mentioned, the ions‏ participate in a series of cellular processes like activating transcription factors (e.g., NF-kB) (Dolmetsch et al., 1998), producing superoxide anions, and secreting proteins and nitric oxide. These can, in turn, disrupt mitochondria harming the cell (Raddassi et al., 1994; Berridge et al., 1998; Xia et al., 2008). Figure 2 shows the schematic representation of the brief cellular toxicity that stemmed from Fe nanoparticles and their bimetallic nanocomposites.

### 4.2 Bioimaging

It is of great value to be able to observe sole molecules in living cells (Pinaud et al., 2010). This is, as it may sound, naturally a hard target, especially in the case of a drug molecule, since the process can inversely affect the molecule’s selectivity or activity due to interactions with the tracer or probe species. Therefore, choosing the functional group which leads to minimum disruptive effects on the functions of the molecules could be critical to be able to use covalent conjugation (Byrne et al., 2007). In few recent cases, the pharmaceutical molecules were conjugated to fluorophores (Uddin et al., 2010), yet this is neither always possible nor free from disadvantages (e.g. photobleaching) (Resch-Genger et al., 2008).

Semiconducting nanoparticles can be used in this case, due to their distinctive optical properties (e.g. long fluorescence lifetimes and controlled sizes) in comparison to common dyes (Michalet et al., 2005; Rosenthal et al., 2011). Surface engineering and bio-functionalization of nanoparticles have added to the potentials of these particles for use as cellular probes applicable in the case of biomolecules (Biju et al., 2010; Zrazhevskiy et al., 2010). The fluorescence signal of nanoparticles can be monitored, *in vivo*, for the determination of properties such as specific targeting and drug release rates.

Zhuo et al. (2019) suggested a new procedure for preparation of iron-doped carbon quantum dots (Fe-CQDs) as bioimaging agents. The procedure constituted hydrothermal carbonization, and the reagents used were ethylenediamine tetraacetic acid (EDTA) salts and ferric nitrate. These quantum dots contain dopamine-bonding Fe sites and luminescent carbon quantum dots (fluorophores). Cell-imaging studies revealed high photostability and low cytotoxicity on the part of the quantum dots, indicating their fitness for biological applications.

### 4.3 Antibacterial Activity

**FIGURE 3:**
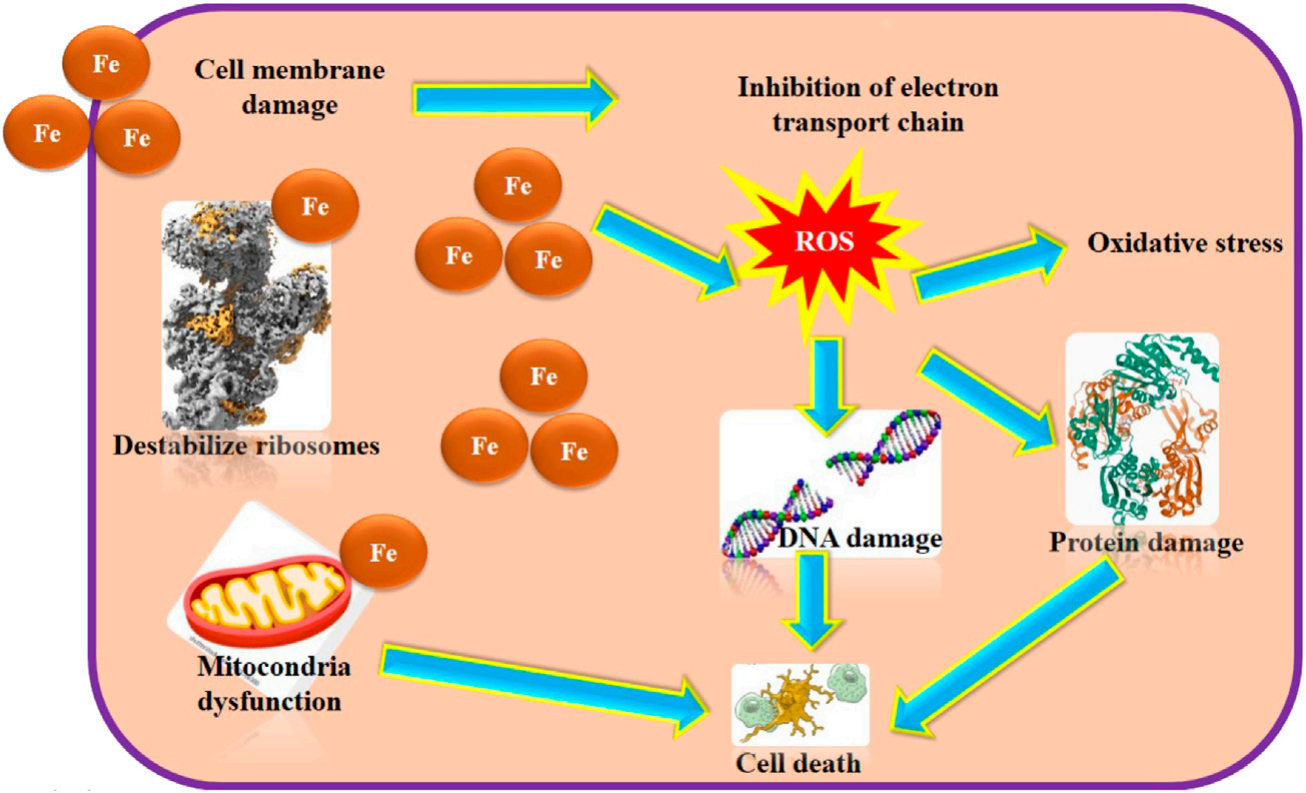
Schematic mechanism for the antibacterial activity by Fe nanoparticles.

Non-toxic nanoparticles of ZnO, Cu and Fe have been prepared and evaluated as an antibacterial material (Table 3). It was found that several factors influence the sensitivity or resistance of bacteria to these nanoparticles. The influences of nanoparticles on pathogenic gene expression hrpE were evaluated through real-time PCR. It was observed that the *Xanthomonas campestris* strain exposed to metallic iron nanoparticles increased the growth rate, and this trend continued with increasing the concentration of these particles. However, increasing the amount of the copper nanoparticles lowered the growth percentage of *Xanthomonas campestris*. The results showed that the expression levels of the pathogenic gene expression hrpE in the case of copper and iron nanoparticles increased 9- and 3-fold (Moradian et al., 2018). The effect of iron nanoparticles on bacteria is a strong function of ROS. Fe nanoparticles are known to sterilize bacteria through exudation, absorption, and complexation. In general, smaller particles have higher antibacterial activity due to their larger surface area. The superoxide radicals, hydrogen peroxide, react to the ROS, damaging deoxyribonucleic acid and cellular proteins leading to the death of the cell. Fe nanoparticles show an antimicrobial activity through generation of ROS under radiation, which in turn kills cells through aerophilous stress on the microorganism. The ROS formed include hydrogen peroxide, superoxide anion radical, and hydroxyl radical. Superoxide anion radicals (^.^O^2−^) undergo reactions with proton ions forming HO^2−^ and then with electrons to form HO^2−^, which next react with protons to form H_2_O_2_. The formed hydrogen peroxide radicals through this multistep reaction reach the deoxyribonucleic acid, and through cell membranes and cellular proteins kill microorganisms. Smaller crystallites form more ROS, and hence nanoparticles are more efficient antibacterial and antimicrobial agents. A second ground for this antibacterial activity is due to the release of Fe^2+^ from the surface of Fe nanoparticles and its interaction with the negatively charged membranes of the microorganism and penetration through the semi-permeable membranes. Based on the results, the nanomaterials directly damage the cell membranes of pathogenic bacteria. A summary of the antibacterial activity by Fe nanoparticles is represented in Figure 3.

**TABLE 3:** Some antibacterial, anticancer, and drug delivery applications of nanoparticles.

Type of nanoparticles	Antibacterial	Cytotoxic	Drug delivery	Reference
Fe-doped ZnO	*Staphylococcus aureus* and *Bacillus subtilis*	—	—	Ravichandran et al. (2017)
Fe-doped ZnO		—	—	Basith et al. (2016)

	*Escherichia coli*, *Pseudomonas aeruginosa*, *Bacillus cereus* ATCC, *Salmonella typhi* MTCC, *Staphylococcus aureus* ATCC, and *Candida albicans* ATCC			
Fe-doped ZnO	*Eschericia coli*	—	—	Gambang (n.d.)
Fe-doped Mn_3_O_4_	*Escherichia coli*	—	—	Belkhedkar and Ubale, (2016)
Fe-doped bioactive glass	*S. aureus* NCIMB-17 and *E. coli* NCIMB-1	Osteosarcoma U_2_OS cells	—	Gupta et al. (2018)
Fe-doped ZnO	*Escherichia coli* (*E.coli*) and *Pseudomonas aeruginosa*	—	—	Kayani et al. (2018)
Fe-doped ZnO	*Escherichia coli* and *Candida albicans*	—	—	Chai et al. (2019)
Fe-doped brushite bone cements	*Staphylococcus aureus* strain, *Escherichia coli* strain, and *Pseudomonas aeruginosa* strain	—	—	Li et al. (2018)
Cu–Fe bimetallic	*S. aureus*, *E. coli*, *and P*. *aeruginosa*	—	—	Bakina et al. (2019)
Fe-doped ZnO	*Staphylococcus aureus* and *Escherichia coli* bacterial strains	MCF 7 cell lines	—	Sekar et al. (2019)
Fe-doped ceria		Neuroblastoma cancer cells and HEK-293 healthy cells	—	Abbas et al. (2015)
Fe-based stents		Mammalian cells	—	Fagali et al. (2015)
Fe and Nd–Fe–B alloy as core carbon shells		Human bone-derived cells	—	Wozniak et al. (2006)
Fe70Pd30 nanotubes	—	IEC-6 cells*	Paracetamol	Rožman et al. (2012)
		SMI-100 cells**		

### 4.4 Drug Delivery

One of the most interactive applications of nanotechnology which is related to cancer treatment is the delivery of pharmaceutical structures to suitable targets (Li et al., 2014). Iron nanoparticles are the major materials which could be employed in drug delivery (Rostami et al., 2021c; Rostami et al., 2021d; Hafezi et al., 2021). Iron nanoparticles have a strong affinity to generate ROS with the photo-Fenton process which helps eradicate tumor tissue. Also, they have been used in gene treatments such as tumor cells. Their distinctive properties such as multi-functionality, considerable volume to surface ration, and the possibility of surface tailoring of iron nanoparticles have led to extensive applications in nanotechnology (Katz and Willner, 2004; Kong et al., 2011).

**FIGURE 4:**
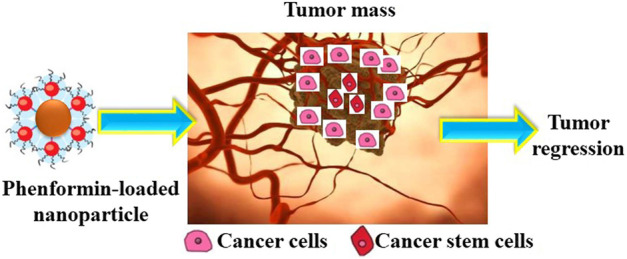
Schematic mechanism for the drug delivery by Fe NPs.

Since Fe nanomaterials have considerable optical properties, they could be used in optical switches, bio-labels, and chemical sensors as well as display systems. Rozman K. Z et al. (Andrade et al., 2020) reported Fe–Pd-based tubular nanostructures. Tests on the magnetic properties of Fe_70_Pd_30_ nanotubes indicated these nanostructures to be ferromagnetic species with a magnetization saturation value of 170 emu g^−1^. This was suggested to make them proper candidates for drug delivery. Li et al. (2019) devised a novel magnetic carrier CNT (Fe)/HA nanocomposite [carbon nanotube (CNT) and hydroxyapatite (HA)] for the targeted delivery of doxorubicin (DOX), using carriers based on a through *in situ* synthesis of carbon nanotubes in the nanoscale hydroxyapatite powder using iron catalysts, followed by chemical modification using folic acid (FA) and chitosan (CS). The synthesis involved the *in situ* self-assembly of Fe, HA, and CNTs into a composite structure followed with acid treatment, which makes the CNTs shorter and homogeneously dispersed. Furthermore, the acid treatment opens the tips of the CNTs and grafts oxygen-containing groups onto them. After functional modification by coating the surface of the tubes with chitosan and folic acid, the composite can be loaded with DOX as a result of π–π stacking and electrostatic adsorption up to an average of 130 wt%. Using a phosphate-buffered saline (PBS) at pH = 5.5, FA-CS-CNT (Fe)/HA released a large quantity of DOX at an average rate of 52 wt% after 72 h, while this value reached only 8 wt% in PBS at pH = 7.4. The respective values of remanence/saturation magnetization, saturation magnetization, and coercive force for the composite were 0.44, 0.88 emu/g, and 668.96 Oe, which reflect the potentials of this composite for drug transport and hence delivery in a strong external magnetic field. Figure 4 shows a summary of the drug delivery caused by Fe nanoparticles schematically.

## 5 Conclusion and Perspectives

Nanobiotechnology is a combination of nanotechnology and biology, which produces eco-friendly products. Today, this field has evolved in the biomedical domain. Fe NPs are a basic element in the body and are an essential nutrient, which play a vital role in catalytic enzymatic reactions inside the cell. Fe NPs are a good strategy for cancer treatment and delivery of pharmaceutical structures to suitable targets. There are various green methods for preparation of Fe NPs using herbal extracts as capping, reducing, and stabilizing agents. The preparation methods can greatly impact the physicochemical and biological characters, and hence, the applications of the products. Single and bimetallic nanoparticles of iron have been used in drug delivery, topic formulations, dressings, and coating of textiles. One of the main reasons for applications of these NPs is due to their antimicrobial ability, which allows for their application in various products ranging from disinfecting agents for different surfaces and medical devices to wound dressings, textiles, and different coatings.

To improve the biomedical applications, researchers are evaluating various approaches for minimizing their toxicity while enhancing their diagnostic and even therapeutic efficiencies. Evaluating the exposure conditions to Fe nanoparticles is not enough. The toxic effects can be due to particle dissolution, agglomeration, and precipitation. These highly depend on size of particles and agglomerates, surface properties, and even exposure mechanisms. Consequently, toxicity assessments should provide a detailed description of exposure conditions as an essential background for the validation of the comparisons. Furthermore, there are not enough toxicity data on low exposure levels, long-term effects, and chronic stress. It is hence critical to concentrate on the long-term effects of chronic exposure to Fe nanoparticles even at low concentrations, which is much closer to real life conditions. Lastly, dissolved Fe^2+^‏ has a key role in toxicity, and there is need for techniques to distinguish the toxicity effects induced by solid Fe nanoparticles and dissolved Fe^2+^‏.
